# Prepregnancy body mass index, gestational weight gain, and birth weight in the BRISA cohort

**DOI:** 10.11606/S1518-8787.2018052000125

**Published:** 2018-04-20

**Authors:** Raina Jansen Cutrim Propp Lima, Rosângela Fernandes Lucena Batista, Marizélia Rodrigues Costa Ribeiro, Cecília Cláudia Costa Ribeiro, Vanda Maria Ferreira Simões, Pedro Martins Lima, Antônio Augusto Moura da Silva, Heloisa Bettiol

**Affiliations:** I Instituto Federal de Educação, Ciência e Tecnologia do Maranhão - Campus Açailândia. Departamento de Ensino. Açailândia, MA, Brasil; II Universidade Federal do Maranhão. Departamento de Saúde Pública. São Luís, MA, Brasil; III Universidade Federal do Maranhão. Departamento de Medicina III. São Luís, MA, Brasil; IV Universidade Federal do Maranhão. Departamento de Odontologia II. São Luís, MA, Brasil; V Universidade Federal do Maranhão. Centro de Ciências Sociais, Saúde e Tecnologia. Imperatriz, MA, Brasil; VI Universidade de São Paulo. Faculdade de Medicina de Ribeirão Preto. Departamento de Puericultura e Pediatria. Ribeirão Preto, SP, Brasil

**Keywords:** Women, Body Mass Index, Pregnancy, Weight Gain, Birth Weight, Maternal and Child Health

## Abstract

**OBJECTIVE:**

To analyze the effects of maternal pre-pregnancy body mass index and weight gain during pregnancy on the baby's birth weight.

**METHODS:**

We conducted a cross-sectional study with 5,024 mothers and their newborns using a Brazilian birth cohort study. In the proposed model, estimated by structural equation modeling, we tested socioeconomic status, age, marital status, pre-pregnancy body mass index, smoking habit and alcohol consumption during pregnancy, hypertension and gestational diabetes, gestational weight gain, and type of delivery as determinants of the baby's birth weight.

**RESULTS:**

For a gain of 4 kg/m^2^ (1 Standard Deviation [SD]) in pre-pregnancy body mass index, there was a 0.126 SD increase in birth weight, corresponding to 68 grams (p < 0.001). A 6 kg increase (1 SD) in gestational weight gain represented a 0.280 SD increase in newborn weight, correponding to 151.2 grams (p < 0.001). The positive effect of pre-pregnancy body mass index on birth weight was direct (standardized coefficient [SC] = 0.202; p < 0.001), but the negative indirect effect was small (SC = -0.076, p < 0.001) and partially mediated by the lower weight gain during pregnancy (SC = -0.070, p < 0.001). The positive effect of weight gain during pregnany on birth weight was predominantly direct (SC = 0.269, p < 0.001), with a small indirect effect of cesarean delivery (SC = 0.011; p < 0.001). Women with a higher pre-pregnancy body mass index gained less weight during pregnancy (p < 0.001).

**CONCLUSIONS:**

The effect of gestational weight gain on the increase in birth weight was greater than that of pre-pregnancy body mass index.

## INTRODUCTION

Birth weight is an indicator of perinatal risk and has been used in epidemiological studies as a representation of fetal nutritional exposure. A secular trend toward increased birth weight related to greater maternal weight has been observed in developed countries^18^. Birth weight reflects the conditions of pregnancy and influences the quality of life, the growth, and the development of the child, as well as childhood morbidity and mortality^22^.

Particularly important among the factors that influence birth weight are the pre-pregnancy and gestational inadequacies of the maternal nutritional status^20^ ^,^ ^27^. Pre-pregnancy overweight and obesity have been associated with gestational hypertension and diabetes, preterm birth, cesarean delivery, and low or high birth weight^22^ ^,^ ^27^. In turn, a low pre-pregnancy body mass index (BMI) has been associated with low birth weight and preterm birth^22^.

A systematic meta-analysis of 45 studies has revealed that low pre-pregnancy BMI increases the risk of infants born small for gestational age with low birth weight, while high pre-pregnancy BMI increases the risk of infants born large for gestational age with high birth weight, macrosomia, and future overweight or obesity^27^. A systematic review of 35 studies has detected strong evidence that excessive gestational weight gain is associated with increased newborn weight (large for gestational age) and that inadequate gestational weight gain is a risk factor for a lower birth weight and for small for gestational age infants^20^. An Argentinian study with 9,613 neonates using multiple (forward stepwise) linear regression models has shown that lower pre-pregnancy BMI was associated with lower birth weight, with no influence of gestational weight gain on birth weight outcome^9^.

Despite the results showing association of pre-pregnancy BMI and weight gain during pregnancy with birth weight, these studies have not investigated whether weight gain during pregnancy is a mediator of the association between pre-pregnancy BMI and birth weight^20^ ^,^ ^27^. Furthermore, most of these studies have used logistic regression analysis with simultaneous adjustment of multiple confounders^9^ ^,^ ^20^ ^,^ ^27^. This type of statistical analysis has been criticized in the literature since it only allows the investigation of associations between the explanatory variables and the outcome, without the possibility of assessing the direct and indirect effects and identifying mediating variables^12^ ^,^ ^25^.

From this perspective, the objective of this study was to respond to the following questions: Is there an association of pre-pregnancy maternal BMI and gestational weight gain with birth weight? Which of the two associations is of greater magnitude? Are these effects direct or do they occur by means of mediators? Is gestational weight gain a mediator of the association between maternal pre-pregnancy BMI and birth weight?

## METHODS

This was a cross-sectional study using data from the 2010 birth cohort in the municipality of São Luís, Maranhão state, Brazil, obtained in the investigation entitled “Etiological factors of preterm birth and consequences of perinatal factors on children's health: birth cohort studies in two Brazilian cities – BRISA”.

The São Luís birth cohort study covered the period from January 1st to December 31st, 2010, including births in public and private hospitals that performed at least 100 deliveries per year. Sample size was calculated based on the number of hospital births that took place in São Luís in 2007, which represented 98% of all births in the city. The method has been detailed elsewhere^21^.

The sample was stratified according to maternity hospital with division proportional to the number of deliveries and was systematic at each maternity. A total of 21,401 births occurred at the maternity hospitals investigated, one third of which were systematically selected from an ordered list of births by hour of occurrence, corresponding to 7,133 births. Of these, 5,475 involved families residing in the municipality for the last three months and therefore eligible for the study. Seventy stillborns, 99 twin births, 239 losses due to early discharge (n = 221) or refusal (n = 18), and 43 discrepant results regarding gestational weight gain were excluded.

We chose to remove puerperae who showed three standard deviations (SD) of total weight gain above or below the mean (12.4 ± 6.76 kg, a weight gain greater than 33 kg or less than -8 kg). Thus, the final study sample consisted of 5,024 births.

The puerperae were preferentially interviewed during the first 24 hours after delivery. The interviewers used a standardized questionnaire and, after obtaining written informed consent, read the questions to the puerperae in order to ensure uniform questions. Information about the newborns was then obtained from the mothers and from the medical records.

The infants were weighed on digital baby scales with 5 g graduations. The infants were weighed wearing no clothing and, if they were crying, their weight was obtained during a deep inspiration. The mothers self-reported their pre-pregnancy weight, weight at the end of pregnancy, and height.

All instruments were calibrated regularly using standard measurements. Premature babies or babies in poor condition at birth, who could not be weighed and measured soon after birth, were reevaluated as soon as their clinical condition allowed.

In the theoretical model proposed, socioeconomic status (SES) was a latent variable that occupied the distal-most position and determined the demographic and nutritional characteristics of the pregnant women, as well as their morbidities, life habits, and type of delivery, which led to the birth weight of their newborns (Figure).

**Figure:**
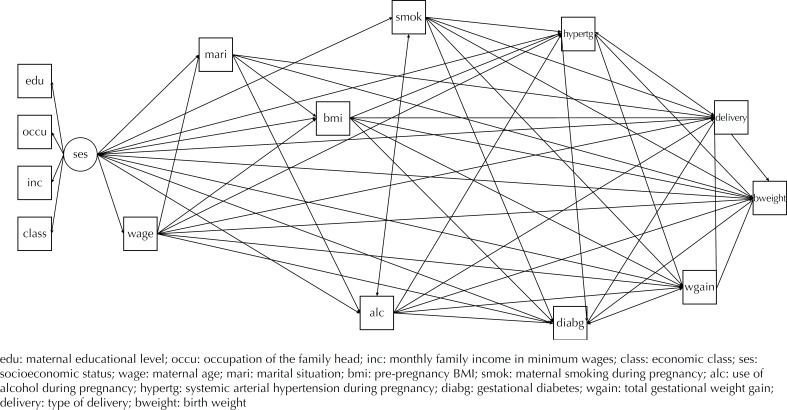
Theoretical model with standardized coefficients for the association of pre-pregnancy BMI and gestational weight gain with birth weight in the BRISA birth cohort study. São Luís, state of Maranhão, Brazil, 2010.

The SES construct was derived from the following variables: maternal schooling (edu – zero to four years, five to eight years, nine to 11 years, 12 years or more of study), occupation of the family head (occu – unskilled manual worker, semi-skilled manual worker, skilled manual worker, office clerk, upper level professonal, and administrators/managers/directors/owners), monthly family income in minimum wages (inc – MW – in 2010 the national minimum wage was R$510.00, approximately US$290.00) (≤ 1, 1 to ≤ 3, 3 to ≤ 5, > 5 or ignored), and economic class (class – D-E, C, A-B). The instrument used to measure economic class was the Brazilian Criteria of Economic Classification created by the Brazilian Association of Research Enterprises (ABEP), which considers the ownership of goods and the schooling of the family head^2^. The economic classes A-B are the more affluent and the D-E classes are the less privileged.

The explanatory variables were pre-pregnancy BMI and gestational weight gain. Pre-pregnancy BMI was determined by dividing the pre-pregnancy weight (kg) by height squared (m), treated in a continuous manner in the model.

Gestational weight gain was calculated as the difference between weight at the end of pregnancy and weight before pregnancy. This variable was used in a continuous manner in the model.

The remaining maternal variables analyzed were maternal age (treated as a continuous numerical variable), marital situation (without a partner, consensual union, or married), smoking during pregnancy (yes or no), alcohol intake during pregnancy (yes or no), type of delivery (vaginal or cesarean), arterial hypertension during pregnancy (yes or no), and gestational diabetes (yes or no), with the last two being self-reported based on the information provided by a physician during prenatal care. The dependent variable was newborn weight, treated as a continuous numerical variable.

Structural equation modeling was used to investigate the association of pre-pregnancy BMI and gestational weight gain with the covariables and their effects on birth weight. This modeling has the advantage of simultaneously handling multiple dependence relationships and it can represent concepts that are not observed (latent variables) in these relationships, modeling the error of measurement in the estimate process^10^.

According to the proposed theory, SES, marital situation (mari), maternal age (wage), pre-pregnancy BMI (bmi), systemic arterial hypertension (hypertg), gestational diabetes (diabg), smoking (smok), alcohol intake (alc), total gestational weight gain (wgain), and type of delivery (delivery) would have a direct effect on birth weight (bweight). In addition, the indirect pathways were proposed starting from SES, maternal age, pre-pregnancy BMI, systemic arterial hypertension, gestational diabetes, smoking, alcohol intake, and gestational weight gain via type of delivery in order to reach the outcome.

We analyzed statistically the data using the Mplus software, version 7. We used the weighted least squares mean and variance adjusted (WLSMV) estimation for continuous and categorical variables. We also used the theta parametrization.

Data regarding some variables were missing (data considered to be ignored in the descriptive analysis), especially pre-pregnancy BMI. However, the WLSMV method for estimation allowed us to the imput these data based on the variables that preceded them in the theoretical model, using frequency analysis and Bayesian analysis^14^.

We considered the following adjustment indices to determine whether the model showed goof fit: a) p-value (*p*) higher than 0.05 for the chi-square test^12^, b) p < 0.05 and an upper limit of the 90% confidence interval of less than 0.08 for the root mean square error of approximation (RMSEA)^25^, c) values higher than 0.95 for the comparative fit index and the Tucker-Lewis Index (CFI/TLI)^25^, and d) values lower than one for the weighted root mean square residual (WRMR)^25^. In the analyses of the standardized estimates for the construction of the latent variable, we considered a factor load of more than 0.5 with p < 0.05 to indicate that the correlation between the variable observed and the construct was of moderately high magnitude^12^.

To obtain suggestions for changes in the initial hypotheses, we calculated modification indices using the MODINDICES command. When the proposed modifications were considered to be plausible from a theoretical viewpoint, a new model was elaborated and analyzed if the value of the modification index was higher than 10^25^.

Total, direct, and indirect effects of the latent variable and of the observed variables were assessed in the final model. An effect was judged to be present when p < 0.05. The mean and SD of the continuous variables were calculated to facilitate the interpretation of the results. The result of the effect on the original metrics of the variable was obtained by multiplying the value of the standardized coefficient of the total effect by the value of the SD of the variable (SC × SD).

The study followed the criteria of Resolution 196/96 of the National Health Council and its complementary clauses. The BRISA project was approved by the Research Ethics Committee of the University Hospital of the Universidade Federal do Maranhão (Opinion 223/2009, Protocol 4771/2008-30).

## RESULTS

The results of the descriptive analysis are presented in Table 1. It is important to note that mean pre-pregnancy BMI was 22.5 (SD = 4) kg/m^2^, mean total gestational weight gain was 12.5 (SD = 6) kg, and mean birth weight was 3.2 (SD = 0.54) kg.

**Table 1:** Socioeconomic and demographic characteristics of mothers and newborns of the BRISA birth cohort study. São Luís, state of Maranhão, Brazil, 2010.

Variable		n	%
Schooling (years)			
	0–4	226	4.5
	5–8	1,113	22.1
	9–11	2,862	57.0
	≥ 12	757	15.1
	Ignored	66	1.3
Marital situation			
	Without a partner	959	19.1
	Consensual union	2,966	59.0
	Married	1,099	21.9
Family income (minimum wages)			
	≤ 1	735	14.6
	1 to ≤ 3	2,033	40.5
	3 to ≤ 5	620	12.3
	> 5	734	14.6
	Ignored	902	18.0
Occupation of the family head			
	Unskilled manual worker	1,470	29.3
	Semi-skilled manual worker	1,851	36.8
	Skilled manual worker	256	5.1
	Office clerk	528	10.5
	Upper level professional	411	8.2
	Administrators/managers/directors/owners	264	5.2
	Ignored	244	4.9
Brazilian Criteria of Economic Classification			
	D–E	1,280	25.5
	C	2,536	50.5
	A–B	909	18.1
	Ignored	299	5.9
Smoking during pregnancy			
	No	4,826	96.1
	Yes	198	3.9
Alcohol during pregnancy			
	No	4,298	85.5
	Yes	726	14.5
Arterial hypertension during pregnancy			
	No	4,205	83.6
	Yes	817	16.3
	Ignored	2	0.1
Gestational diabetes			
	No	4,912	97.8
	Yes	106	2.1
	Ignored	6	0.1
Type of delivery			
	Vaginal	2,668	53.1
	Cesarean	2,356	46.9
Total		5,024	100.0
		Mean	SD
Maternal age (years)		25.1	6
Pre-pregnancy BMI (kg/m^2^)		22.5	4
Gestational weight gain (kg)		12.5	6
Birth weight (kg)		3.2	0.54

The theoretical model showed good fit according to the indicators RMSEA, CFI/TLI, and WRMR, with no plausible suggestion of modification (Table 2).

**Table 2:** Model adjustment indices for birth weight outcome in the BRISA birth cohort study. São Luís, state of Maranhão, Brazil, 2010.

Indices		Model^a^
χ^2^ b		
	Value	117.601
	Degrees of freedom	40
	p	< 0.001
RMSEA		
	Value	0.020
	90%CI	0.016–0.024
	p	0.999
CFI		0.996
TLI		0.991
WRMR		0.788

RMSEA: Root Mean Square Error of Approximation; CFI: Comparative Fit Index; TLI: Tucker-Lewis Index; WRMR: Weighted Root Mean Square Residual

a Initial and final model since there was no suggestion of plausible modification.

b Chi-square test.

The latent variable SES formed a good construct with all indicators having a load factor higher than 0.5 (p < 0.001) (Table 3).

**Table 3:** Standardized coefficients, standard error, and p-value of the direct effects of the indicator variables and construct on birth weight in the BRISA birth cohort study. São Luís, state of Maranhão, Brazil, 2010.

Pathways and estimates		Standardized coefficient	Standard error	p
Latent variable				
SES				
	Maternal schooling	0.729	0.010	< 0.001
	Occupation of the family head	0.667	0.010	< 0.001
	Family income	0.807	0.009	< 0.001
	Economic class	0.874	0.008	< 0.001
Direct effects				
Birth weight				
	SES	-0.166	0.031	< 0.001
	Maternal age	0.018	0.018	0.315
	Marital situation	0.058	0.019	0.002
	Pre-pregnancy BMI	0.202	0.018	< 0.001
	Alcohol intake during pregnancy	0.096	0.039	0.014
	Smoking during pregnancy	-0.155	0.053	0.004
	Arterial hypertension during pregnancy	-0.209	0.029	< 0.001
	Gestational diabetes	0.109	0.043	0.012
	Gestational weight gain	0.269	0.018	< 0.001
	Type of delivery	0.096	0.028	0.001
Maternal age				
	SES	0.340	0.013	< 0.001
Marital situation				
	SES	0.353	0.016	< 0.001
	Maternal age	0.178	0.015	< 0.001
Pre-pregnancy BMI				
	SES	0.051	0.022	0.019
	Marital situation	0.043	0.022	0.048
	Maternal age	0.228	0.016	< 0.001
Alcohol intake during pregnancy				
	SES	-0.058	0.029	0.044
	Marital situation	-0.256	0.030	< 0.001
Smoking during pregnancy				
	SES	-0.265	0.034	< 0.001
	Smoking during pregnancy	0.515	0.034	< 0.001
Arterial hypertension during pregnancy				
	SES	-0.085	0.037	0.019
	Maternal age	0.016	0.023	0.478
	Pre-pregnancy BMI	0.215	0.024	< 0.001
	Alcohol intake during pregnancy	0.063	0.055	0.252
	Smoking during pregnancy	-0.181	0.081	0.026
Gestational diabetes				
	SES	0.175	0.069	0.011
	Maternal age	0.160	0.046	< 0.001
	Pre-pregnancy BMI	0.110	0.041	0.008
	Alcohol intake during pregnancy	-0.052	0.103	0.614
	Smoking during pregnancy	0.019	0.166	0.907
	Arterial hypertension during pregnancy	0.209	0.057	< 0.001
Gestational weight gain				
	SES	0.157	0.028	< 0.001
	Maternal age	-0.007	0.020	0.741
	Pre-pregnancy BMI	-0.260	0.018	< 0.001
	Alcohol intake during pregnancy	-0.067	0.042	0.110
	Smoking during pregnancy	0.047	0.061	0.442
	Arterial hypertension during pregnancy	0.216	0.026	< 0.001
	Gestational diabetes	-0.055	0.049	0.263
Type of delivery				
	SES	0.382	0.030	< 0.001
	Maternal age	0.111	0.020	< 0.001
	Marital situation	0.042	0.022	0.055
	Pre-pregnancy BMI	0.094	0.023	< 0.001
	Alcohol intake during pregnancy	0.018	0.047	0.693
	Smoking during pregnancy	-0.060	0.067	0.364
Arterial hypertension during pregnancy		0.228	0.030	< 0.001
	Gestational diabetes	0.043	0.055	0.431
	Gestational weight gain	0.111	0.021	< 0.001

SES: socioeconomic status; BMI: body mass index

The standardized coefficients of the total and direct effect of indicator and latent variables on birth weight are listed in Table 3. The total direct and indirect effects of pre-pregnancy BMI and weight gain during pregnancy on birth weight can be seen in Table 4.

**Table 4:** Standardized coefficients, standard error, and p-value of the total, direct, and indirect effects of pre-pregnancy BMI and gestational weight gain on birth weight in the BRISA birth cohort study. São Luís, state of Maranhão, Brazil, 2010.

Pathways and estimates		Standardized coefficient	Standard error	p
Total and indirect effects				
Pre-pregnancy BMI				
	Total	0.126	0.015	< 0.001
	Direct	0.202	0.018	< 0.001
	Indirect	-0.076	0.012	< 0.001
	Specific indirect			
	Via gestational weight gain	-0.070	0.007	< 0.001
Via arterial hypertension during pregnancy		-0.045	0.008	< 0.001
Gestational weight gain				
	Total	0.280	0.018	< 0.001
	Direct	0.269	0.018	< 0.001
	Indirect	0.011	0.003	0.001
	Specific indirect			
	Via type of delivery	0.011	0.003	0.001

BMI: body mass index

Pre-pregnancy BMI had positive total and direct effects, revealing that there was an increase of 0.126 SD in birth weight for a gain of 4 kg/m^2^ (1 SD) in pre-pregnancy BMI, corresponding to 68 grams (p < 0.001). Pre-pregnancy BMI also had an indirect and negative effect on birth weight mainly from a gain in gestational weight and on arterial hypertension during pregnancy. There was a negative association between pre-pregnancy BMI and total weight gain at the end of pregnancy and a positive association between pre-pregnancy BMI and hypertension during pregnancy (Table 4).

Weight gain at the end of pregnancy had a positive total effect and a positive direct effect. For each increase of 1 SD in maternal weight gain during pregnancy (6 kg), there was a 0.280 SD increase in birth weight, corresponding to 151.2 grams. Route of delivery also had a small positive indirect effect on weight gain (Table 4).

## DISCUSSION

In this study, higher pre-pregnancy BMI values increase birth weight. The positive effect of weight gain during pregnancy was higher than that of pre-pregnancy BMI. Pre-pregnancy BMI also had a small indirect negative effect on birth weight partially mediated by a lower weight gain and by the presence of arterial hypertension during pregnancy. Women with higher pre-pregnancy BMI gained less weight during pregnancy.

The greater effect of pre-pregnancy BMI was direct and may be explained by the fact that excess maternal weight can trigger a cascade system in which high glucose levels induce insulin production, resulting in increased fetal lipogenesis and excessive fat deposition, giving origin to babies with higher birth weight^26^.

The positive effect of pre-pregnancy BMI on birth weight has been reported in other studies^3^ ^,^ ^11^ ^,^ ^15^ ^,^ ^24^. The Healthy Start study conducted by Starling et al.^23^ on a prospective cohort consisting of women recruited in Colorado, USA, has concluded that maternal BMI was positively associated with neonatal adiposity, using linear regression analysis, with no evidence of an interaction of BMI with gestational weight in the associations with the outcome. The authors have also observed that the increase in maternal BMI was associated with an increase in the fat mass, fat-free mass, and percent body fat of the newborn^23^.

The small indirect negative effect of pre-pregnancy BMI on birth weight mediated by gestational weight gain is a findings that has not been mentioned in other studies^20^ ^,^ ^27^. Women with higher BMI values before pregnancy had a lower gestational weight gain. A possible explanation for this finding is that women with higher pre-pregnancy BMI may have been counseled to control their weight during prenatal care in order to prevent complications for the health of the mother and child. Obese women may benefit from a low weight gain during pregnancy^5^.

The association between maternal overweight and lower gestational weight gain has also been observed among pregnant women seen at public health services in the city of Rio de Janeiro, Brazil, in a prospective study using a multinomial regression model in order to estimate the factors determining insufficient or excessive gestational weight gain and their magnitude and their relationship with adverse maternal-infant outcomes^16^.

A prospective cohort study conducted with 245,526 Swedish pregnant women in order to estimate the effects of low and high gestational weight gain in different ranges of maternal BMI on obstetric and neonatal results has revealed, using linear regression, that obese women with lower gestational weight gain had a reduced risk of pre-eclampsia, cesarean delivery, and the birth of large for gestational age babies. In addition, there was an increased risk of birth of small for gestational age babies among these women^5^. In this study, we could understand this result by structural equation modeling, which demonstrated a small negative indirect effect of pre-pregnancy BMI on birth weight mediated by gestational weight gain.

The relationship between a higher pre-pregnancy BMI and the occurrence of hypertension during pregnancy is already known^6^ ^,^ ^22^. In our study, arterial hypertension during pregnancy had a strong negative effect on birth weight, thus possibly modifying the total effect of pre-pregnancy BMI on the outcome, generating the indirect negative effect observed. A study of a cohort of 1,010 pregnant Ghanaian women conducted in order to assess the perinatal results of pregnancies complicated by hypertensive disorders has reported that women with hypertensive disorders during pregnancy had a higher risk to give birth to babies with low birth weight^4^.

Total weight gain during pregnancy had the highest positive direct effect, as well as an indirect effect. It has been previously demonstrated that there is a direct association between maternal nutrition and newborn weight, with excessive weight gain being considered an independent risk factor for increased birth weight^1^. Some studies have detected an association between high maternal weight gain and increased neonatal adiposity^23^, macrosomia^1^ ^,^ ^20^, and large for gestational age babies^20^.

A positive indirect effect of gestational weight gain via cesarean delivery was also observed. A study of a cohort of 5,564 Brazilian pregnant women has observed that excessive weight gain during pregnancy increases the risk of cesarean delivery and, according to the authors, the indication of cesarean delivery for women with excess weight may be a way to prevent fetal suffering^19^. In turn, cesarean delivery is associated with higher birth weight^13^.

A limitation of our study is that maternal weight and height data were self-reported, thus being subjected to memory bias and underestimation. However, some studies have stated that there is good concordance and validity between reported and measured weight and height information. Thus, self-reported measurements can be used in epidemiological studies^7^ ^,^ ^17^.

A strength of this study is that the sample was random and population based, concerning the population of the city of São Luís, state of Maranhão, Brazil. Another relevant point is the statistical method used to test the association of pre-pregnancy BMI and gestational weight gain with birth weight, i.e., the modeling of stuctural equations. By being able to estimate a series of separate and interdependent multiple regression equations, this method tends to yield more reliable results. Moreover, it allows the estimate of the total, direct, and indirect effects between variables, presenting the ones that are mediating the total effect. In addition, this method yields results that are easy to interpret and allows us to work with initial losses of variables that can be imputed by the method of estimation^8^.

The major and most important findings of this study support the evidence that mothers with higher BMI before pregnancy tend to give birth to heavier babies and that greater gestational weight gain has a greater effect on birth weight than pre-pregnancy BMI. Pregnant women with higher pre-pregnancy BMI and weight gain during pregnancy had newborns with higher birth weights. The greatest positive effect of gestational weight gain on birth weight occurred directly, with little mediation via cesarean delivery. There was also a positive direct effect of pre-pregnancy BMI on birth weight. However, mothers with high pre-pregnancy BMI who gained less weight during pregnancy had children with lower birth weight. These findings support the importance of improving the health care of women of reproductive age by including them in family planning programs with nutritional monitoring and education, so that they will be able to maintain an appropriate nutritional status when they plan to become pregnant and maintain an appropriate weight gain during pregnancy, with a reduced risk of complications for both mother and neonate.

## References

[1] Amorim MMR, Leite DFB, Gadelha TGN, Muniz AGV, Melo ASO, Rocha AM (2009). Fatores de risco para macrossomia em recém-nascidos de uma maternidade-escola no nordeste do Brasil. Rev Bras Ginecol Obstet.

[2] Associação Brasileira de Empresas de Pesquisa (2015). Critério de Classificação Econômica Brasil 2012.

[3] Bhattacharya S, Campbell DM, Liston WA, Bhattacharya S (2007). Effect of Body Mass Index on pregnancy outcomes in nulliparous women delivering singleton babies. BMC Public Health.

[4] Browne JL, Vissers KM, Antwi E, Srofenyoh EK, Van der Linden EL, Agyepong IA (2015). Perinatal outcomes after hypertensive disorders in pregnancy in a low resource setting. Trop Med Int Health.

[5] Cedergren M (2006). Effects of gestational weight gain and body mass index on obstetric outcome in Sweden. Int J Gynaecol Obstet.

[6] Doherty DA, Magann EF, Francis J, Morrison JC, Newnham JP (2006). Pre-pregnancy body mass index and pregnancy outcomes. Int J Gynaecol Obstet.

[7] Fonseca MJM, Faerstein E, Chor D, Lopes CS (2004). Validade de peso e estatura informados e índice de massa corporal: estudo pró-saúde. Rev Saude Publica.

[8] Gamborg M, Andersen PK, Baker JL, Budtz-Jorgensen E, Jorgensen T, Jensen G (2009). Life course path analysis of birth weight, childhood growth, and adult systolic blood pressure. Am J Epidemiol.

[9] Grandi CA (2003). Relación entre la antropometría materna y la ganancia de peso gestacional con el peso de nacimiento, y riesgos de peso bajo al nacer, pequeño para la edad gestacional y prematurez en una población urbana de Buenos Aires. Arch Latinoam Nutr.

[10] Hair JF, Black WC, Babin BJ, Anderson RE, Tatham RL (2005). Análise multivariada de dados.

[11] Khashan AS, Kenny LC (2009). The effects of maternal body mass index on pregnancy outcome. Eur J Epidemiol.

[12] Kline RB (2011). Principles and practice of structural equation modeling.

[13] Li H, Ye R, Pei L, Ren A, Zheng X, Liu J (2014). Caesarean delivery, caesarean delivery on maternal request and childhood overweight: a Chinese birth cohort study of 181 380 children. Pediatr Obes.

[14] Muthén LK, Muthén BO (c1998-2010). Mplus: statistical analysis with latent variables: user's guide.

[15] Najafian M, Cheraghi M (2012). Occurrence of fetal macrosomia rate and its maternal and neonatal complications: a 5-year cohort study. ISRN Obstet Gynecol.

[16] Rodrigues PL, Oliveira LC, Brito AS, Kac G (2010). Determinant factors of insufficient and excessive gestational weight gain and maternal-child adverse outcomes. Nutrition.

[17] Schmidt MI, Duncan BB, Tavares M, Polanczyk CA, Pellanda L, Zimmer PM (1993). Validity of self-reported weight: a study of urban Brazilian adults. Rev Saude Publica.

[18] Scientific Advisory Committee on Nutrition (2011). The influence of maternal, fetal and child nutrition on the development of chronic disease in later life.

[19] Seligman LC, Duncan BB, Branchtein L, Gaio DSM, Mengue SS, Schmidt MI (2006). Obesity and gestational weight gain: cesarean delivery and labor complications. Rev Saude Publica.

[20] Siega-Riz AM, Viswanathan M, Moos MK, Deierlein A, Mumford S, Knaack J (2009). A systematic review of outcomes of maternal weight gain according to the Institute of Medicine recommendations: birthweight, fetal growth, and postpartum weight retention. Am J Obstet Gynecol.

[21] Silva AAM, Batista RFL, Simões VMF, Thomaz EBAF, Ribeiro CCC, Lamy F (2015). Changes in perinatal health in two birth cohorts (1997/1998 and 2010) in São Luís, Maranhão State, Brazil. Cad SaudePublica.

[22] Stang J, Huffman LG (2016). Position of the Academy of Nutrition and Dietetics: obesity, reproduction, and pregnancy outcomes. J Acad Nutr Diet.

[23] Starling AP, Brinton JT, Glueck DH, Shapiro AL, Harrod CS, Lynch AM (2015). Associations of maternal BMI and gestational weight gain with neonatal adiposity in the Healthy Start study. Am J Clin Nutr.

[24] Trombe KSD (2014). Relação entre IMC pré-gestacional e tamanho do recém-nascido na coorte de conveniência de 2010 de Ribeirão Preto.

[25] Wang J, Wang X (2012). Structural equation modeling: applications using Mplus.

[26] Worthington-Roberts BS, Williams SR (1997). Nutrition in pregnancy and lactation.

[27] Yu Z, Han S, Zhu J, Sun X, Ji C, Guo X (2013). Pre-pregnancy body mass index in relation to infant birth weight and offspring overweight/obesity: a systematic review and meta-analysis. Plos One.

